# Temporal Control of Morphogenic Factor Expression Determines Efficacy in Enhancing Regeneration

**DOI:** 10.3390/plants10112271

**Published:** 2021-10-23

**Authors:** Juan H. Gonzalez, Joseph S. Taylor, Kelsey M. Reed, R. Clay Wright, Bastiaan O. R. Bargmann

**Affiliations:** 1 School of Plant and Environmental Sciences, Virginia Polytechnic Institute and State University (Virginia Tech), Blacksburg, VA 24061, USA; juanhg@vt.edu (J.H.G.); jstaylo8@vt.edu (J.S.T.); kelseyreed@vt.edu (K.M.R.); 2 Department of Biological Systems Engineering, Virginia Polytechnic Institute and State University (Virginia Tech), Blacksburg, VA 24061, USA; wrightrc@vt.edu

**Keywords:** plant regeneration, morphogenic factors, conditional expression, auxin signaling

## Abstract

Background: Regeneration of fertile plants from tissue culture is a critical bottleneck in the application of new plant breeding technologies. Ectopic overexpression of morphogenic factors is a promising workaround for this hurdle. Methods: Conditional overexpression of *WUS* and *ARF5*Δ was used to study the effect of timing the overexpression of these morphogenic factors during shoot regeneration from root explants in Arabidopsis. In addition, their effect on auxin-signaling activation was examined by visualization and cytometric quantification of the DR5:GFP auxin-signaling reporter in roots and protoplasts, respectively. Results: The induced expression of both *WUS* and *ARF5*Δ led to an activation of auxin signaling in roots. Activation of auxin signaling by *WUS* and *ARF5*Δ was further quantified by transient transformation of protoplasts. Ectopic overexpression of both *WUS* and *ARF5*Δ enhanced regeneration efficiency, but only during the shoot-induction stage of regeneration and not during the callus-induction stage. Conclusions: The overexpression of *WUS* and *ARF5*Δ both lead to activation of auxin signaling. Expression during the shoot-induction stage is critical for the enhancement of shoot regeneration by *WUS* and *ARF5*Δ.

## 1. Introduction

Crop trait improvement through genetic modification holds increasing potential to efficiently address the challenges faced in sustainable food production around the world, as foundational research identifies more genetic targets and technical advances in gene-editing tools allow for more precise manipulation of the plant genome. However, the regeneration of fertile plants from transformed or edited cells is a major bottleneck in the application of these new plant breeding technologies [1]. Numerous species, or genotypes within species, are recalcitrant to tissue culture and plant regeneration.

Ectopic overexpression of morphogenic factors is a promising tool to help overcome recalcitrance in regeneration and accelerate and broaden the applicability of new plant breeding technologies [2]. Such factors can reprogram plant cells in tissue culture and push them towards embryogenesis or shoot meristem development. However, the temporal control of their overexpression is crucial to the utility of these tools, as constitutive expression can lead to undesirable developmental phenotypes that negate their effectiveness.

WUSCHEL (WUS) is a homeodomain transcription factor that can promote vegetative-to-embryonic transition when overexpressed [3]. Constitutive or inducible overexpression of Arabidopsis (*Arabidopsis thaliana*) *WUS* has been shown to enhance regeneration efficiency in recalcitrant crops, cotton (*Gossypium hirsutum*) and coffee (*Coffea canephora*), respectively [4,5].

An irrepressible form of MONOPTEROS/AUXIN RESPONSE FACTOR 5 (ARF5Δ) has been shown to enhance shoot regeneration in Arabidopsis [6]. ARF5 is a transcription factor in the auxin signal transduction pathway and ARF5Δ lacks the C-terminal PB1 domain that is required for interaction with the Aux/IAA repressor proteins. *ARF5*Δ expression leads to hyperactivation of the transcriptional auxin response [7]. In these cases, the expression of *ARF5*Δ was driven by the native *ARF5* promoter.

In Arabidopsis, root explants can readily be induced to form shoots by a short (one week) incubation on callus induction medium (CIM; high auxin/cytokinin ratio) followed by transfer to shoot induction medium (SIM; low auxin/cytokinin ratio). Here, we have made use of conditional expression of *WUS* and *ARF5*Δ together with the root-to-shoot regeneration assay in Arabidopsis to assess the effect of the timing of expression of these morphogenic factors (during incubation on CIM and/or SIM) on regeneration efficiency. In addition, we examined the ability of *WUS* and *ARF5*Δ constructs to induce the expression of the auxin-responsive DR5:GFP reporter gene [8] in roots and root-derived protoplasts.

## 2. Results

### 2.1. Ectopic Expression of WUS and ARF5*Δ* Induces DR5:GFP in Roots

In order to assess the effect of ectopic expression of *WUS* and *ARF5*Δ on auxin signaling activity, one-week-old seedlings with the DR5:GFP reporter gene and β-estradiol-inducible expression cassettes for *WUS* or *ARF5*Δ (XVE:WUS and XVE:ARF5Δ, respectively) were transferred to plates with varying amounts of β-estradiol (0, 0.1, or 1.0 µM). The expression of the reporter was visualized microscopically after one day of treatment. As a control for the conditional expression, plants carrying a β-estradiol-inducible *GFP* expression cassette (XVE:GFP) were also transferred to β-estradiol plates.

**Figure 1:**
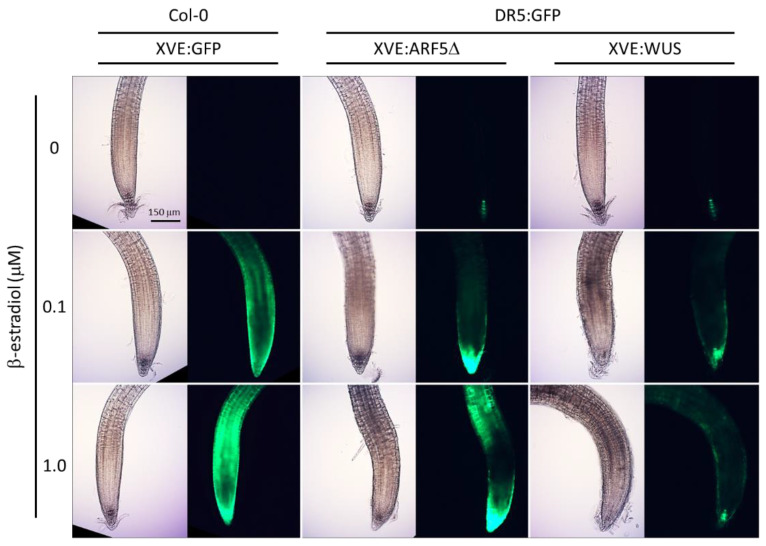
Ectopic overexpression of *WUS* or *ARF5*Δ induces DR5:GFP in root tips. One-week-old seedlings were transferred to plates with varying amounts of β-estradiol and imaged in brightfield and GFP fluorescence after 24 h. Wild-type (Col-0) plants were transformed with XVE:GFP (left panel). Plants carrying DR5:GFP were transformed with XVE:ARF5Δ and XVE:WUS (center and right panels, respectively). The scale bar represents 150 µm.

While there was no GFP expression in XVE:GFP seedlings transferred to 0 µM control plates, their transfer to 0.1 and 1.0 µM β-estradiol plates led to a proportional increase in GFP expression throughout the roots (Figure 1). The XVE:WUS and XVE:ARF5Δ lines showed typical DR5:GFP expression in the quiescent center and columella cells when transferred to control plates. Transfer of these lines to plates with β-estradiol led to a marked increase in and ectopic expression of the DR5:GFP reporter in XVE:ARF5Δ root tips (Figure 1). DR5:GFP expression also increased and appeared ectopically in the XVE:WUS seedlings transferred to β-estradiol plates, although to a lesser extent than the XVE:ARF5Δ line (Figure 1). These results show that induced expression of *WUS* and *ARF5*Δ both lead to ectopic activation of auxin signaling in seedling roots.

### 2.2. Transient Expression of ARF5*Δ* and WUS Activates Auxin Signaling in Root Protoplasts

Transient expression and cytometric analysis of protoplasts allow for quantitative analysis of DR5:GFP reporter gene activation and can give an indication whether the activation seen in roots is context dependent or cell autonomous. Therefore, pBeaconRFP vectors [9] were constructed carrying either *ARF5*Δ, *WUS,* or a *GUS* control and used to transform root protoplasts from a DR5:GFP reporter line. Using the pBeaconRFP system, the concurrent expression of the RFP positive fluorescent selection marker with the gene-of-interest permits the cytometric isolation of the successfully transformed cells for quantification of the GFP signal. Following the transformation procedure, the protoplast suspensions were treated with 50 nM indole-3-acetic acid (or mock-treated) and analyzed 24 h later.

**Figure 2:**
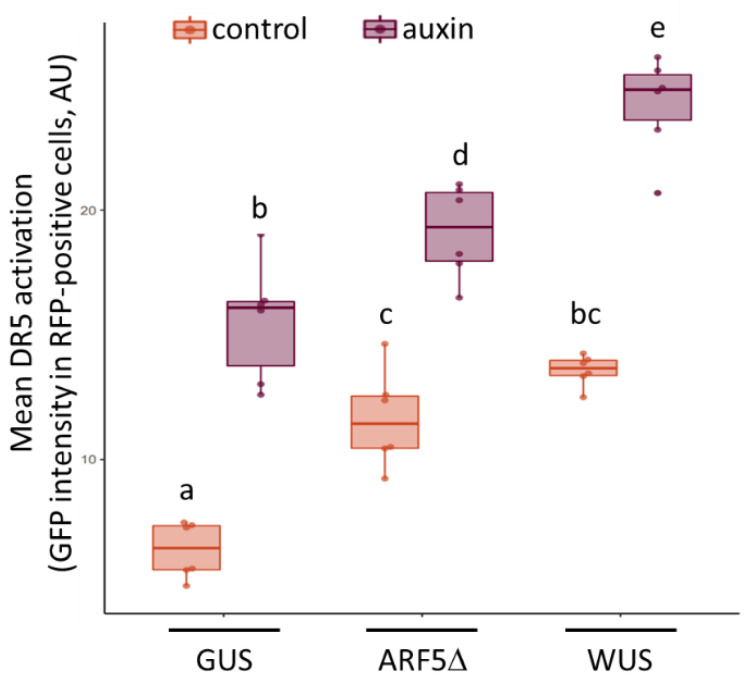
DR5:GFP activation in protoplasts transiently transformed with *WUS* or *ARF5*Δ. Protoplasts derived from the roots of one-week-old DR5:GFP seedlings were transformed with pBeaconRFP vectors containing a *GUS* control, *ARF5*Δ, or *WUS*. Protoplasts were treated with 50 nM IAA, or mock treated, for 24 h and GFP intensity (arbitrary units) in the RFP-positive cells was quantified by flow cytometry. Treatments were performed in triplicate on two independent transformations; statistical significance was assayed by two-way ANOVA followed by a Tukey post hoc test; letters indicate homogeneous subsets (*p* < 0.05), *n* = 6.

The protoplasts transformed with the *GUS* control showed a basal level of DR5:GFP activity that increased significantly when treated with auxin (Figure 2). As expected, protoplasts transformed with the *ARF5*Δ construct displayed a basal level and an auxin-induced increase in DR5:GFP that were both significantly higher than in the respective GUS controls (two-way ANOVA, treatment *p* = 7.03 × 10^−16^, construct *p* = 2.49 × 10^−11^, interaction *p* = 0.11; Tukey post hoc test, *p* < 0.05). Expression of *WUS* increased DR5:GFP expression similarly to *ARF5*Δ. Interestingly, the auxin-induced increase in DR5:GFP expression was higher still in protoplasts transformed with the *WUS* construct (Figure 2). These results indicate that both *WUS* and *ARF5*Δ expression can lead to a cell-autonomous increase in auxin signaling.

### 2.3. Effects of Conditional Expression of WUS and ARF5*Δ* on Regeneration Efficiency

In order to examine the effects of the timing of the overexpression of *WUS* and *ARF5*Δ on enhancing shoot regeneration efficiency, root explants were transferred to CIM and subsequent SIM with or without supplemented β-estradiol. In addition to the XVE:WUS and XVE:ARF5Δ lines, XVE:GFP was included as a control. The ratio of explants that had or had not formed shoots was monitored over the three weeks on SIM.

**Figure 3:**
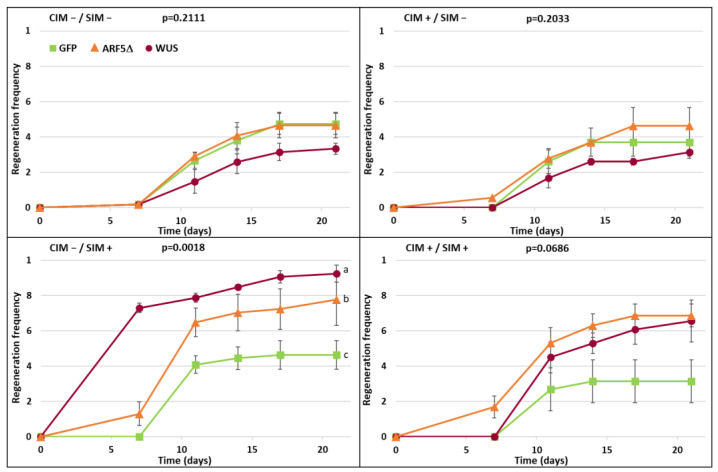
Efficiency of shoot regeneration in plants expressing *GFP*, *WUS,* or *ARF5*Δ. Root segments from one-week-old seedlings of plants carrying XVE:GFP, XVE:WUS, or XVE:ARF5Δ were induced to form shoots by transfer to CIM for one week followed by transfer to SIM for three weeks. CIM/SIM were supplemented with 0.1 µM β-estradiol (+) or equivalent volume of vehicle as a control (−). Regeneration was measured as the fraction of explants forming shoots over the three weeks on SIM. The *p* value for a one-way ANOVA of the area-under-the-curve comparing overexpressed genes is presented for each treatment. Error bars indicate standard error of the mean and letters indicate homogeneous subsets in post hoc testing using the Holm method (*p* < 0.05), *n* = 3.

**Figure 4:**
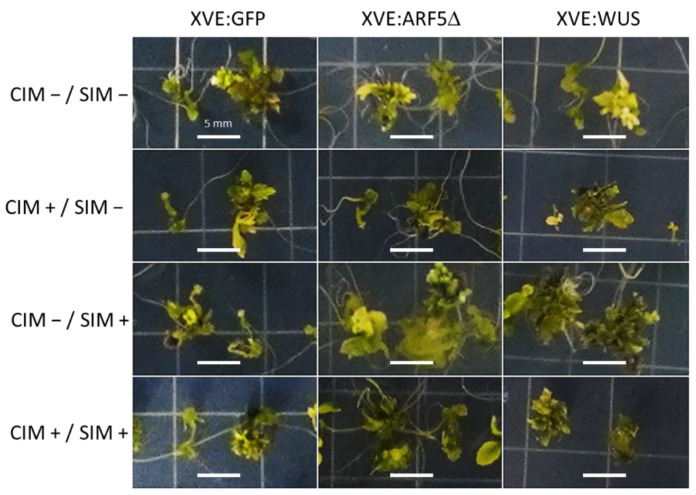
Morphology of shoot regeneration in plants expressing *GFP*, *WUS,* or *ARF5*Δ. Root segments from one-week-old seedlings of plants carrying XVE:GFP, XVE:WUS, or XVE:ARF5Δ were induced to form shoots by transfer to CIM for one week followed by transfer to SIM. Explants were transferred either to control (−) plates or plates supplemented with 0.1 µM β-estradiol (+). Two representative regenerating explants were imaged after four weeks on SIM. The scale bars represent 5 mm.

Without inclusion of β-estradiol in either CIM or SIM, only about half of the explants had formed shoots after three weeks (Figure 3). Similar results were obtained when β-estradiol was included only in the CIM plates. β-estradiol induction during incubation only on SIM led to a marked increase in regeneration efficiency in both XVE:WUS and XVE:ARF5Δ lines, with XVE:WUS showing the greatest increase in efficiency. This difference was statistically significant (one-way ANOVA *p* = 0.0018, Holm post hoc test *p* < 0.05). Overexpression of *WUS* or *ARF5*Δ during both CIM and SIM incubation resulted in some increase in regeneration efficiency, but not as strong as treatment during SIM alone and not significantly different from the *GFP* control (Figure 3). Looking at the morphology of the regenerated shoots, induction of *WUS* and *ARF5*Δ during incubation on SIM led to a more robust shoot production (Figure 4). These results demonstrate that the timing of expression of morphogenic factors WUS and ARF5Δ determines their effect on enhanced regeneration efficiency.

## 3. Discussion

### 3.1. Morphogenic Factors WUS and ARF5*Δ* Both Enhance Auxin Signaling

Expression of *ARF5*Δ under the native *ARF5* promoter was previously reported to lead to ectopic DR5:GFP expression in leaves [7]. We show here that overexpression of *ARF5*Δ also leads to enhanced and ectopic expression of the DR5:GFP reporter in seedling roots (Figure 1). This is in line with expectations, as ARF5Δ lacks the PB1 domain that facilitates interaction with the Aux/IAA repressors of auxin signaling. Hence, the irrepressible ARF5Δ can bind to the auxin-response elements in the DR5 promoter, and presumably also to those in the promoters of other auxin-responsive genes, and can activate expression even in cells where Aux/IAAs are present.

Interestingly, overexpression of *WUS* also led to enhanced and ectopic expression of the DR5:GFP reporter in seedling roots, albeit to a lesser extent than *ARF5*Δ (Figure 1). This may be unexpected, as overexpression of an activatable WUS-GR fusion protein has been reported to maintain a low auxin signaling output in shoot stem cells, as measured by a DR5 reporter [10]. It seems, therefore, that the effect of *WUS* expression on auxin signaling is context dependent and has different outcomes in the shoot and root meristems. Other *WUS* related genes are known to be expressed in auxin-signaling maxima and enhance auxin signaling when overexpressed, e.g., WUSCHEL-RELATED HOMEOBOX 5 expression in the root meristem [11,12].

When measured in isolated cells, using transient transformation of protoplasts, *ARF5*Δ increased the expression of the DR5:GFP reporter, both basally and in response to auxin treatment (Figure 2). *WUS* overexpression also activated auxin signaling in protoplasts. The fact that WUS induced DR5:GFP activation in roots to a lesser extent than ARF5Δ, whereas the opposite was observed in the protoplast assay, may be attributed to differences in overexpression levels between the two systems and constructs or differences in the post-transcriptional regulation of *ARF5*Δ and *WUS*, e.g., [13]. The results in protoplasts indicate that WUS activates auxin signaling cell autonomously and that the activation seen in roots is likely not due to alterations in auxin transport and accumulation but rather a consequence of (in)direct activation of auxin signaling or, potentially, enhanced auxin biosynthesis.

The conditional overexpression of both *WUS* and the auxin biosynthesis gene *YUCCA1* have recently independently been shown to enhance regeneration from protoplasts [14,15]. In future work, it will be interesting to examine whether direct and specific activation of auxin signaling with an irrepressible ARF (like ARF5Δ or other A-class ARFs) could perform in a similar manner.

### 3.2. Timing the Overexpression of Morphogenic Factors to Enhance Shoot Regeneration

Ectopic overexpression of morphogenic factors to enhance shoot regeneration efficiency has been employed successfully in several studies. However, undesirable developmental phenotypes can result from constitutive overexpression of such factors. Several approaches can be undertaken to address this problem; i.e., excision of the morphogenic gene [16], use of a promoter that is only expressed during the early stages of regeneration [17], transient overexpression by avoiding genomic integration [18], or conditional expression/activation [19]. The latter example used the BABY BOOM transcription factor fused to the glucocorticoid receptor, which allowed for ligand-induced nuclear translocation and activation of the factor.

Here, we use the β-estradiol-inducible XVE system [20] to ensure *WUS* or *ARF5*Δ are only overexpressed during the regeneration process and do not lead to developmental abnormalities during normal plant growth. Furthermore, we used this system to investigate whether expression during the callus-induction stage and/or the shoot-induction stage of regeneration would most effectively enhance regeneration efficiency. Results clearly demonstrate that expression only during the shoot-induction stage best enhances regeneration efficiency, both in the case of *WUS* and *ARF5*Δ (Figure 3 and Figure 4). Expression only during the callus-induction stage had no effect and looked just like the non-induced control. Whereas there appeared to be some enhanced regeneration efficiency when induced both during the callus- and shoot-induction stages, this was not statistically significant and markedly less than when expression occurred only during shoot induction.

It is interesting that a similar effect was observed for both *WUS* and *ARF5*Δ, suggesting both may enhance regeneration efficiency through a related process. Indeed, a *WUS* reporter gene was seen to be expressed during the shoot-induction stage of cotyledon regeneration in *ARF5*Δ plants [6], indicating that *ARF5*Δ may act upstream of *WUS* in enhancing the shoot regeneration process. During shoot regeneration from root explants, incubation on CIM leads to pericycle reactivation and the formation of lateral root meristems, while subsequent incubation on SIM leads to a transition of those lateral root meristems to shoot meristems [21]. The results presented here suggest that both WUS and ARF5Δ promote that latter transition and do not enhance regeneration through the influence on reactivation of the xylem-pole pericycle in the early stages of regeneration.

## 4. Materials and Methods

### 4.1. Plant Material and Growth Conditions

All genotypes are in the Columbia (Col-0) background of Arabidopsis. XVE:ARF5Δ was generated as described previously [22]. Briefly, the first 2382 nucleotides of *ARF5* (encoding the first 794 amino acids and lacking the C-terminal PB1 domain) were cloned into the β-estradiol-inducible, gateway-compatible vector pMDC7 [23]; this vector was used to transform plants carrying the DR5rev::GFP reporter (ABRC stock CS9361) using *Agrobacterium tumefaciens* strain GV3101. XVE:WUS was generated similarly, from a pENTR-D-TOPO plasmid (ABRC stock TOPO-U14-E11) into pMDC7, which was used to transform plants carrying the DR5rev::GFP reporter. XVE:GFP was generated by transforming wild-type Col-0 plants with pMDC7 carrying *eGFP_ER* cloned from pK7GWIWG2D(II) [24]. Seeds were sterilized by 5 min incubation with 70% (*v*/*v*) ethanol followed by 10 min incubation with 20% (*v*/*v*) household bleach and triple rinsing with sterile water. Seeds were plated on square 10 × 10 cm plates and incubated in a plant growth chamber (Percival, Perry, IA, USA) at 22 °C with an 18 h light/6 h dark regime at 75 µmol m^−2^ s^−1^ PAR.

### 4.2. Microscopy

GFP expression in seedling roots was visualized using an inverted epifluorescence microscope (IX81, Olympus, Waltham, MA, USA) equipped with a Moticam 5+ camera (Motic Instruments, San Antonio, TX, USA). Exposure time for the XVE:GFP line was 1/15 that of the time used for DR5:GFP visualization in the XVE:WUS and XVE:ARF5Δ lines.

### 4.3. Protoplast Isolation, Transformation, and Treatment

Roots of one-week-old seedlings were harvested and placed into a gently shaking 250 mL flask with 50 mL enzymolysis solution (1.25% [*w*/*v*] Cellulase R-10 [Yakult Pharmaceutical Industry Co., Somerset, NJ, USA], 0.3% [*w*/*v*] Macerozyme R-10 [Yakult Pharmaceutical Industry Co., Somerset, NJ, USA], 0.4 M mannitol, 20 mM MES, 20 mM KCl, 10 mM CaCl_2_, 0.1% [*w*/*v*] bovine serum albumin; pH was adjusted to 5.7 with Tris-HCl pH 7.5) for 4 h. The protoplast solution was filtered through a 40 µm Falcon cell strainer (VWR, Radnor, PA, USA), divided over 15 mL conical tubes, and centrifuged for 5 min at 500× *g*. Cells were resuspended in enzymolysis buffer (no enzymes added), counted with a hemacytometer, and centrifuged again. Cells were washed once with transfection solution (0.4 M mannitol, 15 mM MgCl_2_ hexahydrate, 4 mM MES; pH was adjusted to 5.7 with KOH), centrifuged again, and resuspended in transfection solution with a final density of 4 * 10^6^ protoplasts mL^−1^. Conical tubes (15 mL) were prepared for each transfection with 25 µg of plasmid DNA and 250 µL of protoplasts in transfection solution. Protoplasts were transformed with the pBeaconRFP vector [9] carrying *WUS*, *ARF5*Δ, or a *GUS* (β-glucuronidase) control. Next, 250 µL PEG solution (40% [*w*/*v*] PEG 1500, 0.4 M mannitol, 0.1 M CaCl_2_) was added, and the suspension was mixed by flicking the tube repeatedly. Suspensions were incubated for 1 min, after which the protoplasts were washed with 15 mL enzymolysis buffer, centrifuged, and resuspended in 1 mL enzymolysis buffer. Protoplast suspensions were divided in 96-well plates (150 µL per well), treated with 50 nM indole-3-acetic acid (or mock treated), and incubated overnight in the dark at room temperature. Two independent transformations were performed for each construct and treatments were performed in triplicate.

### 4.4. Flow Cytometry

The GFP intensity in RFP-positive cells was quantified using a C6 flow cytometer (Accuri, Minneapolis, MN, USA) with 488 nm excitation, forward-scatter and side-scatter, 515/20 nm emission for GFP, and 585/40 nm emission for RFP. Gates were set to identify live cells (based on the forward-scatter–side-scatter population expressing DR5:GFP), isolate cells expressing RFP, and then quantify the GFP intensity (arbitrary units) in those cells. Statistical significance was assessed by two-way ANOVA and subsequent Tukey post hoc testing to identify homogeneous subsets.

### 4.5. Regeneration Assay

The regeneration assay was based on previously published methods [25]. Briefly, seeds were plated on 1/2MS1 medium (2.2 g/L Murashige and Skoog basal medium [MilliporeSigma, Burlington, MA, USA], 1% [*w*/*v*] sucrose, pH 5.8) with 1.5% (*w*/*v*) agar in rows of 20–30. Plates were placed vertically in the growth chamber. After one week, 5 mm root explants were excised 5 mm above the root tip and placed on CIM plates (2.2 g/L Murashige and Skoog basal salt mixture [MilliporeSigma, Burlington, MA, USA], 1x Gamborg’s vitamin solution [MilliporeSigma, Burlington, MA, USA], 2% [*w*/*v*] glucose, pH 5.8, 0.2 µM kinetin, 2.2 µM 2,4-Dichlorophenoxyacetic acid, +/− 0.1 µM β-estradiol), 18 explants per plate. After one week, explants were transferred to SIM plates (2.2 g/L Murashige and Skoog basal salt mixture [MilliporeSigma, Burlington, MA, USA], 1x Gamborg’s vitamin solution [MilliporeSigma, Burlington, MA, USA], 2% [*w*/*v*] glucose, pH 5.8, 0.9 µM indole-3-acetic acid, 5 µM 2-isopentenyladenine, +/− 0.1 µM β-estradiol). The number of explants forming shoots was monitored over the next three weeks. Regeneration efficiency was scored as (the number of explants forming shoots)/(total explants) and the assay was performed in triplicate. Statistical significance was assessed by one-way ANOVA of the area-under-the-curve and post hoc testing by the Holm method [26].

## Data Availability

Not applicable.

## References

[1] Atkins P.A., Voytas D.F. (2020). Overcoming Bottlenecks in Plant Gene Editing. Curr. Opin. Plant Biol..

[2] Gordon-Kamm B., Sardesai N., Arling M., Lowe K., Hoerster G., Betts S., Jones T. (2019). Using Morphogenic Genes to Improve Recovery and Regeneration of Transgenic Plants. Plants.

[3] Zuo J., Niu Q.-W., Frugis G., Chua N.-H. (2002). The WUSCHEL Gene Promotes Vegetative-to-Embryonic Transition in Arabidopsis. Plant J..

[4] Bouchabké-Coussa O., Obellianne M., Linderme D., Montes E., Maia-Grondard A., Vilaine F., Pannetier C. (2013). Wuschel Overexpression Promotes Somatic Embryogenesis and Induces Organogenesis in Cotton (*Gossypium Hirsutum* L.) Tissues Cultured in Vitro. Plant Cell Rep..

[5] Arroyo-Herrera A., Ku Gonzalez A., Canche Moo R., Quiroz-Figueroa F.R., Loyola-Vargas V.M., Rodriguez-Zapata L.C., Burgeff D′Hondt C., Suárez-Solís V.M., Castaño E. (2008). Expression of WUSCHEL in Coffea Canephora Causes Ectopic Morphogenesis and Increases Somatic Embryogenesis. Plant Cell Tiss. Organ. Cult..

[6] Ckurshumova W., Smirnova T., Marcos D., Zayed Y., Berleth T. (2014). Irrepressible MONOPTEROS/ARF5 Promotes de Novo Shoot Formation. New Phytol..

[7] Krogan N.T., Ckurshumova W., Marcos D., Caragea A.E., Berleth T. (2012). Deletion of MP/ARF5 Domains III and IV Reveals a Requirement for Aux/IAA Regulation in Arabidopsis Leaf Vascular Patterning. New Phytol..

[8] Ulmasov T., Murfett J., Hagen G., Guilfoyle T.J. (1997). Aux/IAA Proteins Repress Expression of Reporter Genes Containing Natural and Highly Active Synthetic Auxin Response Elements. Plant Cell.

[9] Bargmann B.O., Birnbaum K.D. (2009). Positive Fluorescent Selection Permits Precise, Rapid, and in-Depth Overexpression Analysis in Plant Protoplasts. Plant Physiol..

[10] Ma Y., Miotk A., Šutiković Z., Ermakova O., Wenzl C., Medzihradszky A., Gaillochet C., Forner J., Utan G., Brackmann K. (2019). WUSCHEL Acts as an Auxin Response Rheostat to Maintain Apical Stem Cells in Arabidopsis. Nat. Commun..

[11] Sarkar A.K., Luijten M., Miyashima S., Lenhard M., Hashimoto T., Nakajima K., Scheres B., Heidstra R., Laux T. (2007). Conserved Factors Regulate Signalling in Arabidopsis Thaliana Shoot and Root Stem Cell Organizers. Nature.

[12] Savina M.S., Pasternak T., Omelyanchuk N.A., Novikova D.D., Palme K., Mironova V.V., Lavrekha V.V. (2020). Cell Dynamics in WOX5-Overexpressing Root Tips: The Impact of Local Auxin Biosynthesis. Front. Plant Sci..

[13] Snipes S.A., Rodriguez K., DeVries A.E., Miyawaki K.N., Perales M., Xie M., Reddy G.V. (2018). Cytokinin Stabilizes WUSCHEL by Acting on the Protein Domains Required for Nuclear Enrichment and Transcription. PLoS Genet..

[14] Xu M., Du Q., Tian C., Wang Y., Jiao Y. (2021). Stochastic Gene Expression Drives Mesophyll Protoplast Regeneration. Sci. Adv..

[15] Sakamoto Y., Kawamura A., Suzuki T., Segami S., Maeshima M., Polyn S., Veylder L.D., Sugimoto K. (2021). Transcriptional Activation of Auxin Biosynthesis Drives Developmental Reprogramming of Differentiated Cells. bioRxiv.

[16] Lowe K., Wu E., Wang N., Hoerster G., Hastings C., Cho M.-J., Scelonge C., Lenderts B., Chamberlin M., Cushatt J. (2016). Morphogenic Regulators Baby Boom and Wuschel Improve Monocot Transformation. Plant Cell.

[17] Lowe K., La Rota M., Hoerster G., Hastings C., Wang N., Chamberlin M., Wu E., Jones T., Gordon-Kamm W. (2018). Rapid Genotype “Independent” Zea Mays L. (Maize) Transformation via Direct Somatic Embryogenesis. Vitro Cell. Dev. Biol.-Plant.

[18] Richael C.M., Kalyaeva M., Chretien R.C., Yan H., Adimulam S., Stivison A., Weeks J.T., Rommens C.M. (2008). Cytokinin Vectors Mediate Marker-Free and Backbone-Free Plant Transformation. Transgenic. Res..

[19] Heidmann I., de Lange B., Lambalk J., Angenent G.C., Boutilier K. (2011). Efficient Sweet Pepper Transformation Mediated by the BABY BOOM Transcription Factor. Plant Cell Rep..

[20] Zuo J., Niu Q.W., Chua N.H. (2000). An Estrogen Receptor-based Transactivator XVE Mediates Highly Inducible Gene Expression in Transgenic Plants. Plant J..

[21] Atta R., Laurens L., Boucheron-Dubuisson E., Guivarc’h A., Carnero E., Giraudat-Pautot V., Rech P., Chriqui D. (2009). Pluripotency of Arabidopsis Xylem Pericycle Underlies Shoot Regeneration from Root and Hypocotyl Explants Grown in Vitro. Plant J. Cell Mol. Biol..

[22] Wu M.-F., Yamaguchi N., Xiao J., Bargmann B., Estelle M., Sang Y., Wagner D. (2015). Auxin-Regulated Chromatin Switch Directs Acquisition of Flower Primordium Founder Fate. Elife.

[23] Curtis M.D., Grossniklaus U. (2003). A Gateway Cloning Vector Set for High-Throughput Functional Analysis of Genes in Planta. Plant Physiol..

[24] Karimi M., Inzé D., Depicker A. (2002). GATEWAY^TM^ Vectors for Agrobacterium-Mediated Plant Transformation. Trends Plant Sci..

[25] Meng W.J., Cheng Z.J., Sang Y.L., Zhang M.M., Rong X.F., Wang Z.W., Tang Y.Y., Zhang X.S. (2017). Type-B Arabidopsis Response Regulators Specify the Shoot Stem Cell Niche by Dual Regulation of Wuschel. Plant Cell.

[26] Aickin M., Gensler H. (1996). Adjusting for Multiple Testing When Reporting Research Results: The Bonferroni vs Holm Methods. Am. J. Public Health.

